# Dataset for off-grid micro-hydroelectric community system under drought conditions

**DOI:** 10.1016/j.dib.2020.105717

**Published:** 2020-05-19

**Authors:** Kathleen B. Aviso, Isidro Antonio V. Marfori, Raymond R. Tan, Aristotle T. Ubando

**Affiliations:** a Chemical Engineering Department, De La Salle University, Manila, Philippines; b Mechanical Engineering Department, De La Salle University, Manila, Philippines

**Keywords:** Polygeneration systems, off-grid systems, micro-hydroelectric powerplant

## Abstract

This submission contains the complete balanced process matrix of an off-grid community system primarily powered by a micro-hydroelectric powerplant. The system is meant to provide the needs of the community for electricity, potable water and ice. The system also considers the provision of a diesel engine generator set as a back-up to provide electricity. The data serves as inputs to simulate the performance of the system under different drought scenarios. The data provided here is in support of the co-submitted article of Aviso et al. [1].

Specifications table

**Table:** 

Subject	Energy
Specific subject area	This work focuses on the design and utilization of a micro-hydroelectric system for an off-grid community utility system.
Type of data	Numerical data was obtained from optimizing the off-grid utility system presented in Aviso et al. [1] using the Fuzzy P-graph model. Data included here are presented in tabular form with their corresponding process diagrams illustrated in Figures in P-graph representation. Data were generated using P-graph Studio version:5.2.3.2.
How data were acquired	Data was obtained by extracting the information from the fuzzy P-graph optimization model developed by Aviso et al. [1] and implemented in P-graph Studio version: 5.2.3.2.
Data format	Raw Analyzed
Parameters for data collection	Parameters include the process coefficients of units included in the polygeneration system, the demand for identified product streams given as a range, and the level of drought given as a percent reduction in available water from the baseline. Material streams of interest are electricity, clean water and ice as products, and diesel fuel as raw material.
Description of data collection	Data was collected from the Fuzzy P-graph model implemented by Aviso et al. [1]. The information provided includes the capacity of the process units, the flow of streams in between process units, and the net demand output and resource input in each scenario.
Data source location	The data in the simulations are hypothetical but based on a representative off-grid village. Characteristics of the micro-hydroelectric turbine are based on prototypes developed in De La Salle University.
Data accessibility	Complete data for simulations are given in this data article. Additional information on the micro-hydroelectric turbine are available from the authors upon request.
Related research article	Aviso, K.B., Marfori, I.A.V., Tan, R.R. and Ubando, A.T., 2020, Optimizing Abnormal Operations of Off-Grid Community Utility Systems with Fuzzy P-graph, Energy, 202, 117725.

## Value of the data

• The data provides details on the performance of the off-grid community utility system during different drought scenarios to allow readers to replicate or modify the simulations.
• Data illustrates how unavailability of water resources can impact the performance of a micro-hydroelectric based off-grid system.
• Information will be useful for decision-makers and community planners in the design of renewable energy systems which are affected by changes in climate.
• Data can be used to conduct further analysis on similar off-grid utility systems which may include other types of renewable energy or other process units.

## 1 Data Description

The data was obtained using the optimization model described in Aviso et al. [1] and modelled in P-graph Studio Version: 5.2.3.2. The model was implemented in a laptop using an Intel® Core^TM^ i7-6500U CPU @ 2.50 GHz processor. The input parameters include the process performance of the different units included in the off-grid polygeneration system, the demand range for the product streams of interest as well as level of drought experienced by the system. The system consists of a micro-hydroelectric powerplant (MHP) which generates electricity using 2 different turbines (MHT1 and MHT2), an ice plant which manufactures ice, an ultra-filtration water treatment (UFWT) facility to generate potable water and a back-up diesel generator set to supply electricity. The different scenarios examined are summarized in Table 1 where a drought level from 0 to 50% is implemented when there is no available diesel generator set for back-up (Scenarios 1 to 6). If the drought level reaches 60%, no feasible solution can be found if there is no available diesel generator set. Scenarios 7 to 16 on the other hand examines drought scenarios from 10% to 100% with an available diesel generator set. The over-all level of satisfaction (λ) achieved by the system, which is a function of net output of ice, electricity and clean water and the net input of diesel, are also shown in Table 1. Detailed information regarding the optimal solutions for the different scenarios presented are shown in tables and figures as follows.

**Table 1:** Summary of scenarios considered

Scenario	Drought level	Available water (t/d)	Availability of Diesel Generator	Over-all λ achieved
1	0 %	52,550	No	1.00
2	10 %	47,295	No	0.80
3	20 %	42,040	No	0.60
4	30 %	36,785	No	0.40
5	40 %	31,530	No	0.20
6	50 %	26,275	No	0.005
7	10%	52,550	Yes	0.91
8	20%	47,295	Yes	0.81
9	30%	42,040	Yes	0.72
10	40%	36,785	Yes	0.63
11	50%	31,530	Yes	0.53
12	60 %	21,020	Yes	0.44
13	70 %	15,765	Yes	0.35
14	80 %	10,510	Yes	0.25
15	90 %	5,255	Yes	0.07
16	100 %	0	N/A	Not feasible

**Fig. 1:**
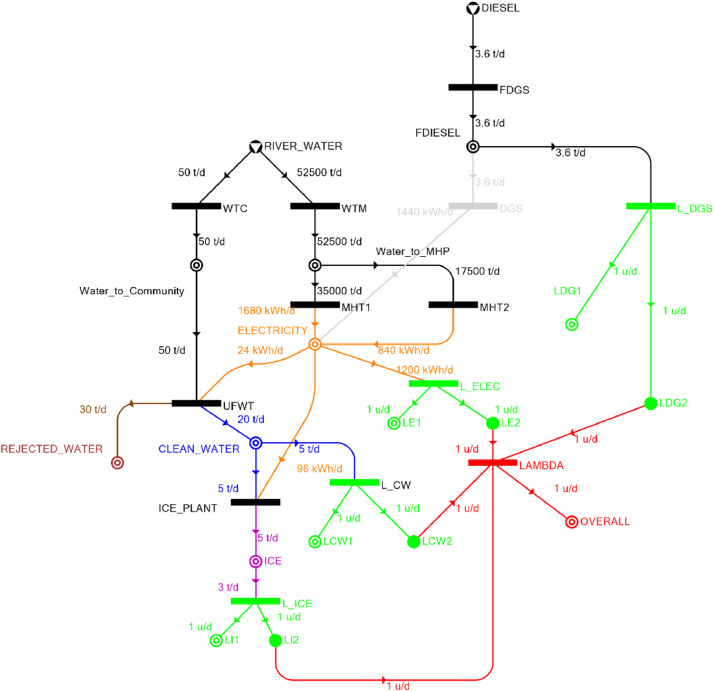
P-graph representation of micro-hydroelectric system during normal operating conditions.

**Fig. 2:**
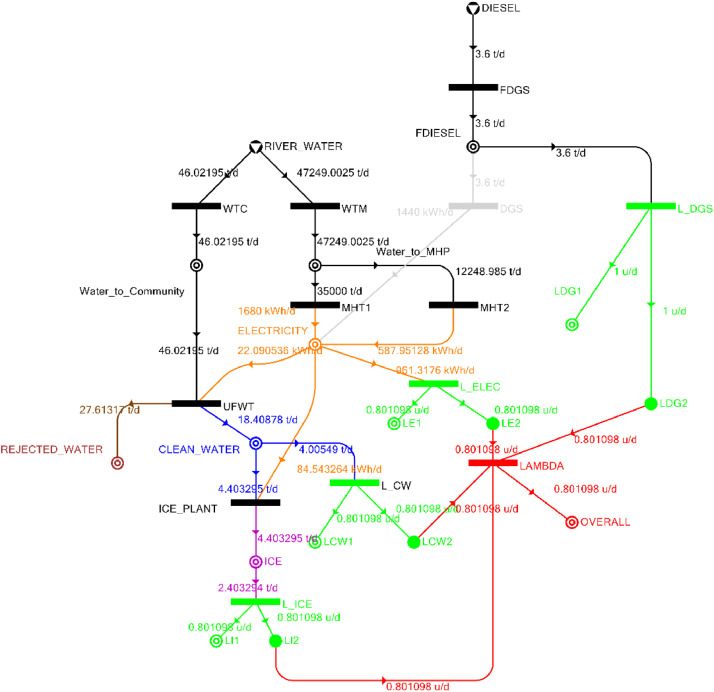
P-graph representation of micro-hydroelectric system at 10% drought level.

**Fig. 3:**
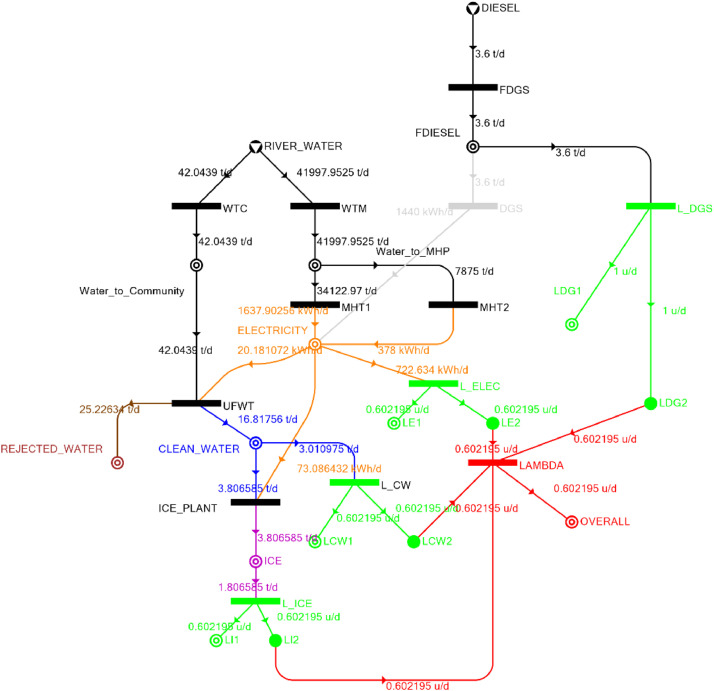
P-graph representation of micro-hydroelectric system at 20% drought level.

**Fig. 4:**
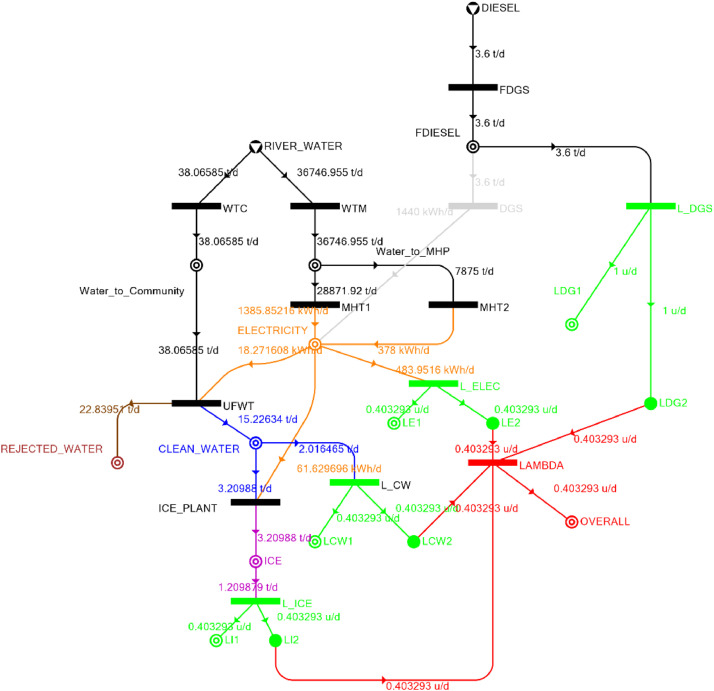
P-graph representation of micro-hydroelectric system at 30% drought level.

**Fig. 5:**
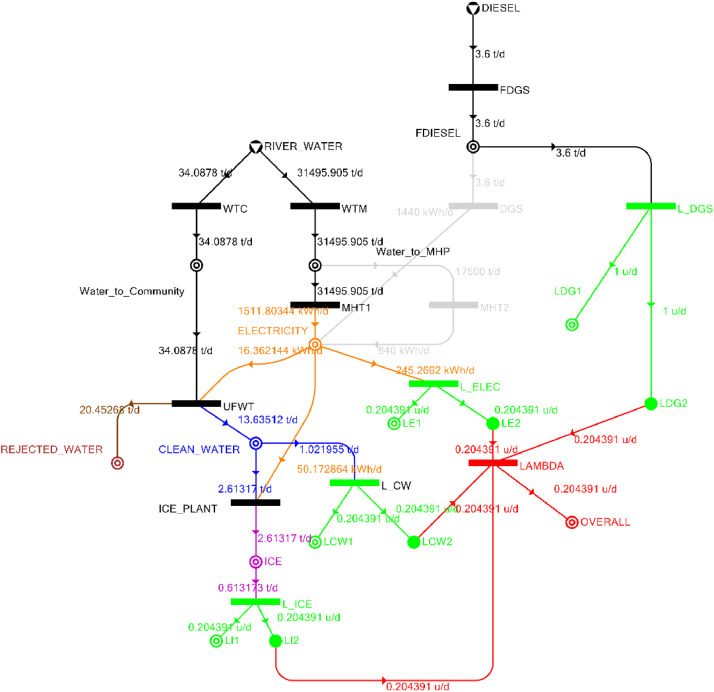
P-graph representation of micro-hydroelectric system at 40% drought level.

**Fig. 6:**
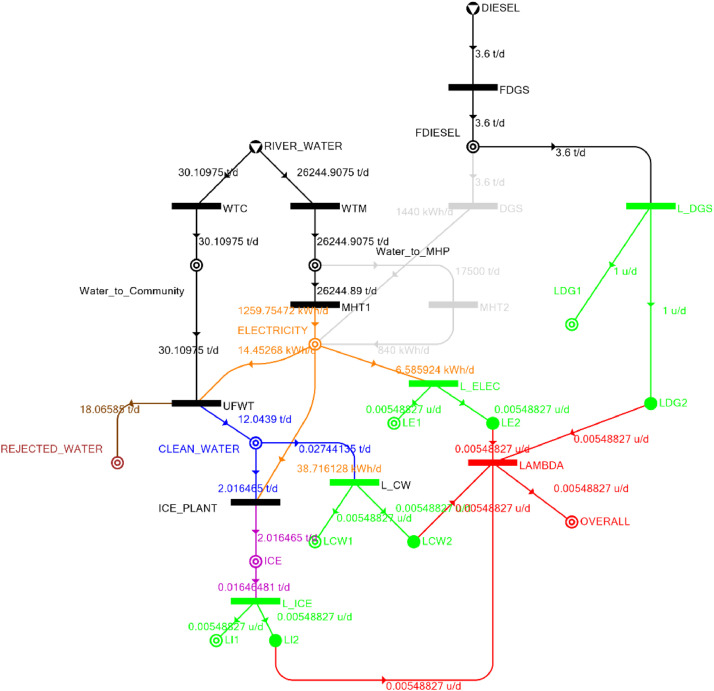
P-graph representation of micro-hydroelectric system at 50% drought level.

**Fig. 7:**
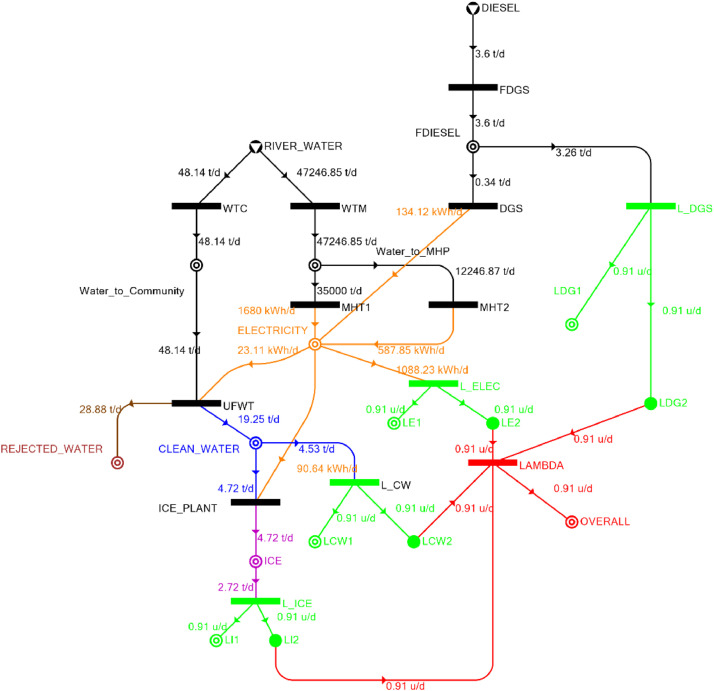
P-graph representation of micro-hydroelectric system at 10% drought level with Diesel Generator.

**Fig. 8:**
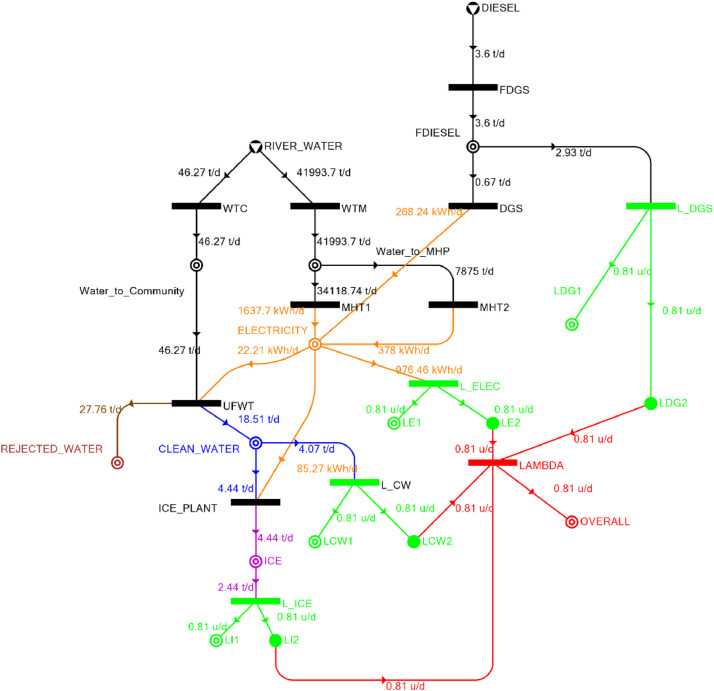
P-graph representation of micro-hydroelectric system at 20% drought level with Diesel Generator.

**Fig. 9:**
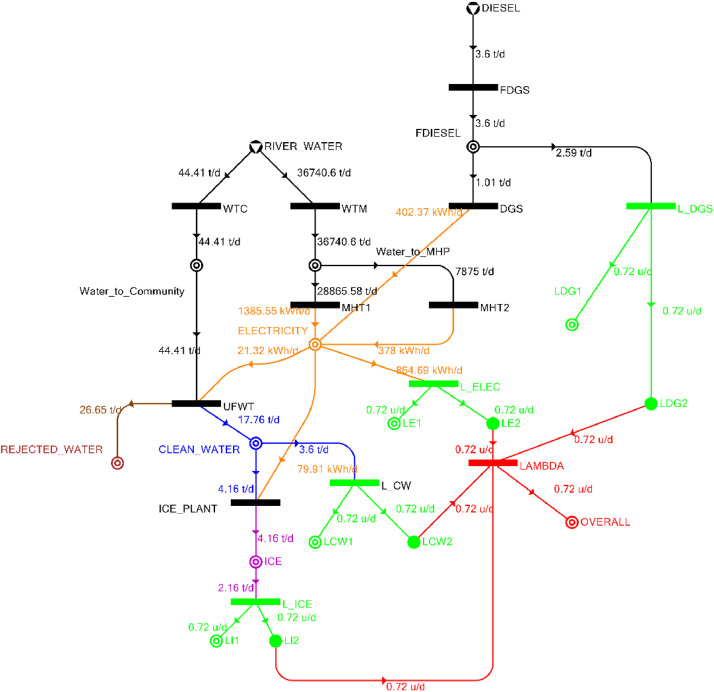
P-graph representation of micro-hydroelectric system at 30% drought level with Diesel Generator.

**Fig. 10:**
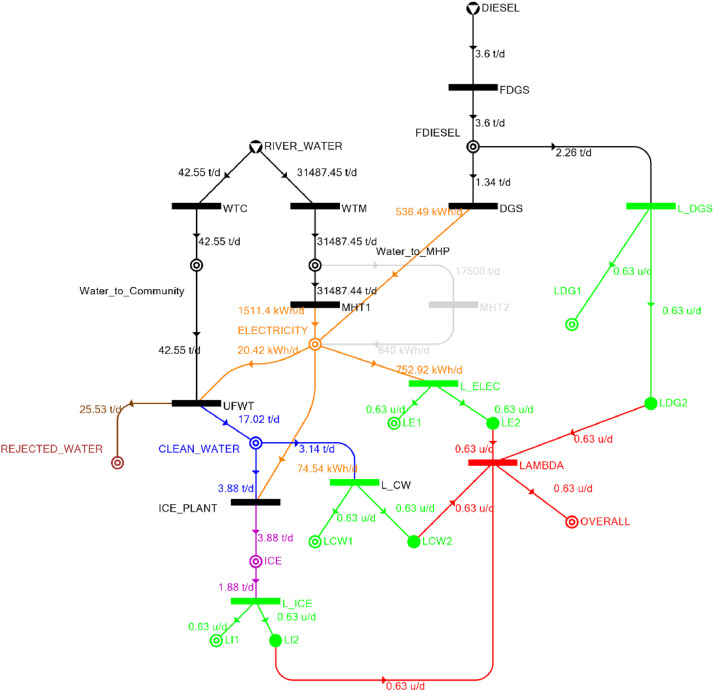
P-graph representation of micro-hydroelectric system at 40% drought level with Diesel Generator.

**Fig. 11:**
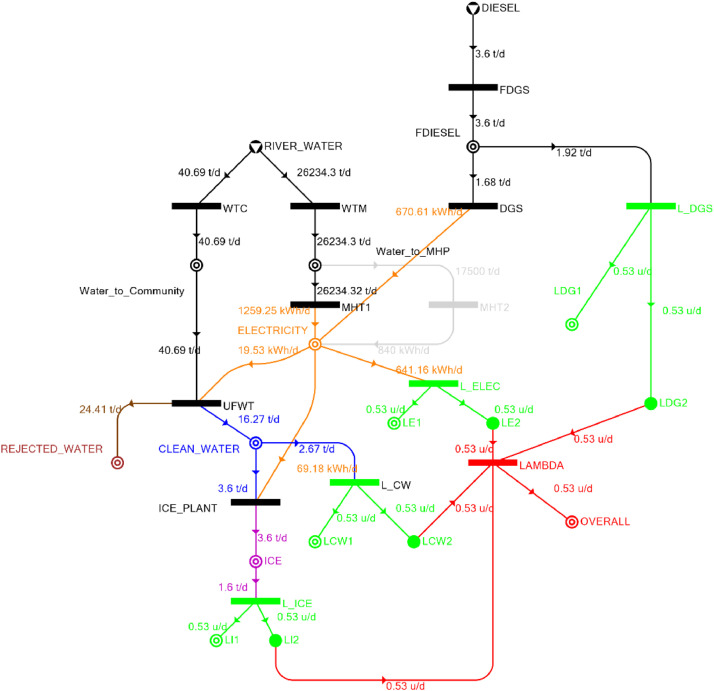
P-graph representation of micro-hydroelectric system at 50% drought level with Diesel Generator.

**Fig. 12:**
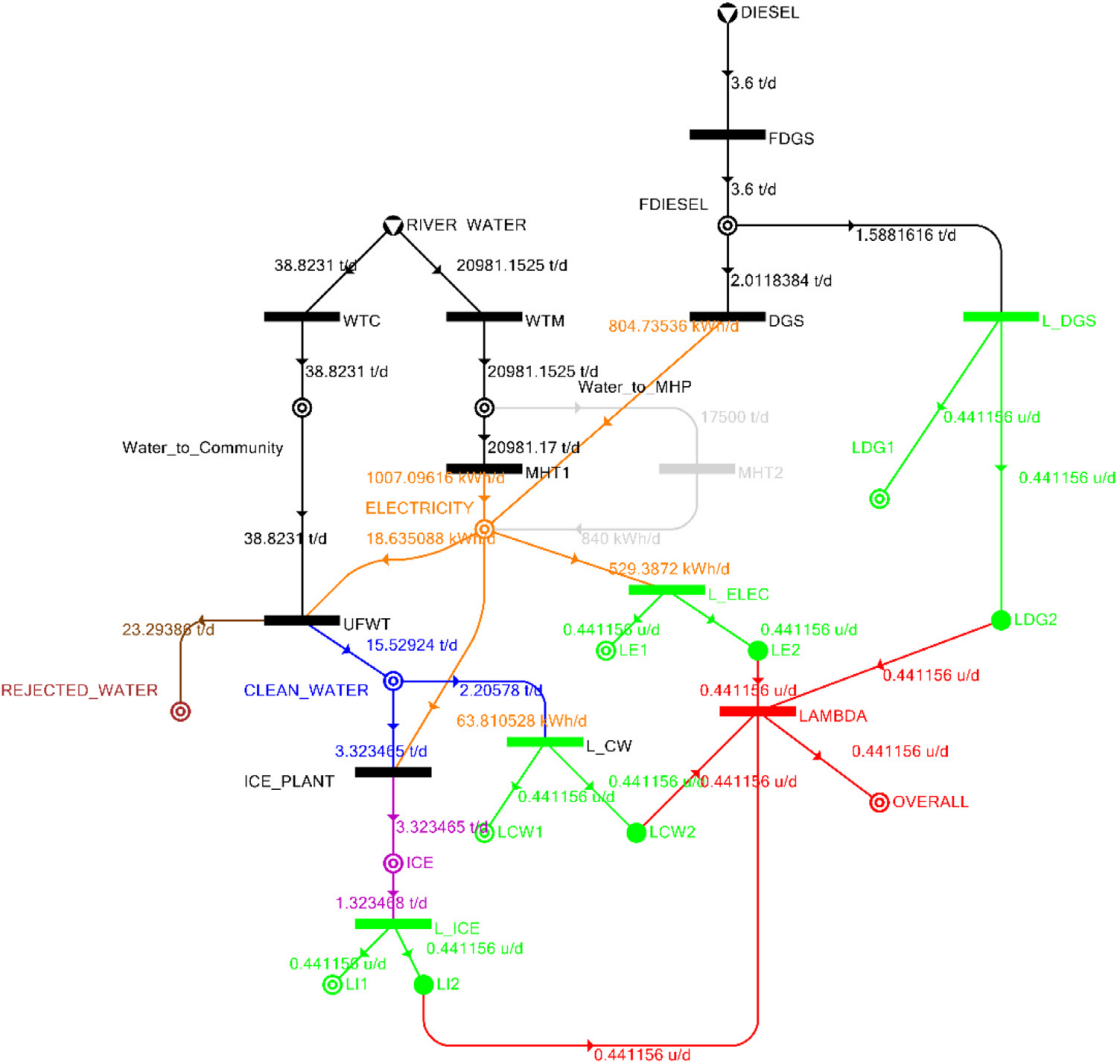
P-graph representation of micro-hydroelectric system at 60% drought level with Diesel Generator.

**Fig. 13:**
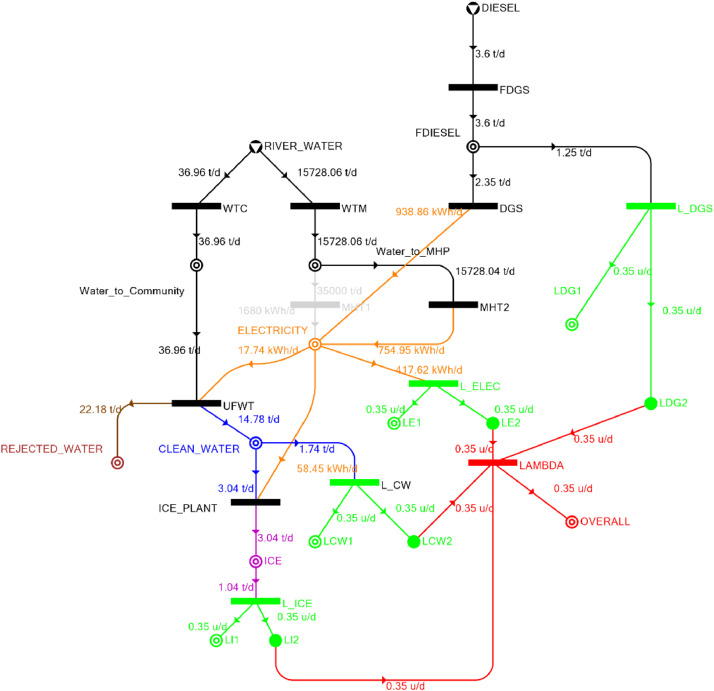
P-graph representation of micro-hydroelectric system at 70% drought level with Diesel Generator.

**Fig. 14:**
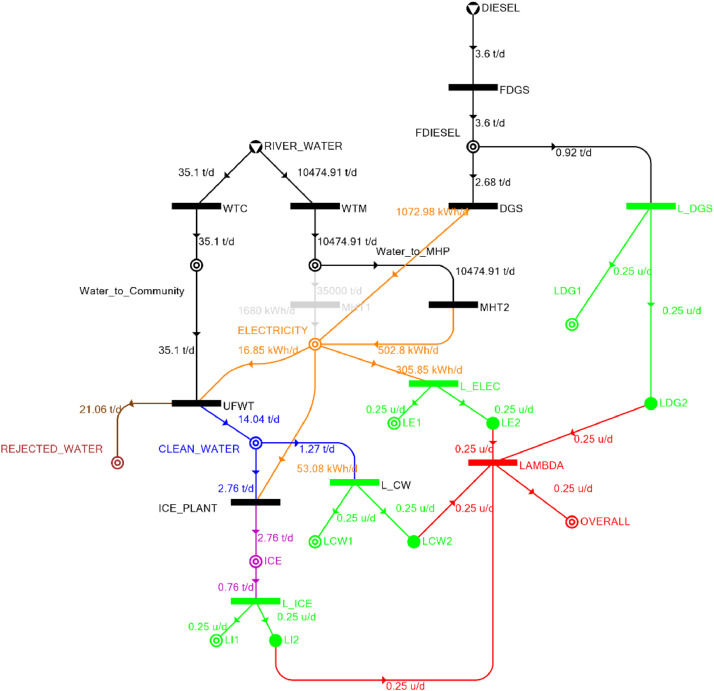
P-graph representation of micro-hydroelectric system at 80% drought level with Diesel Generator.

**Fig. 15:**
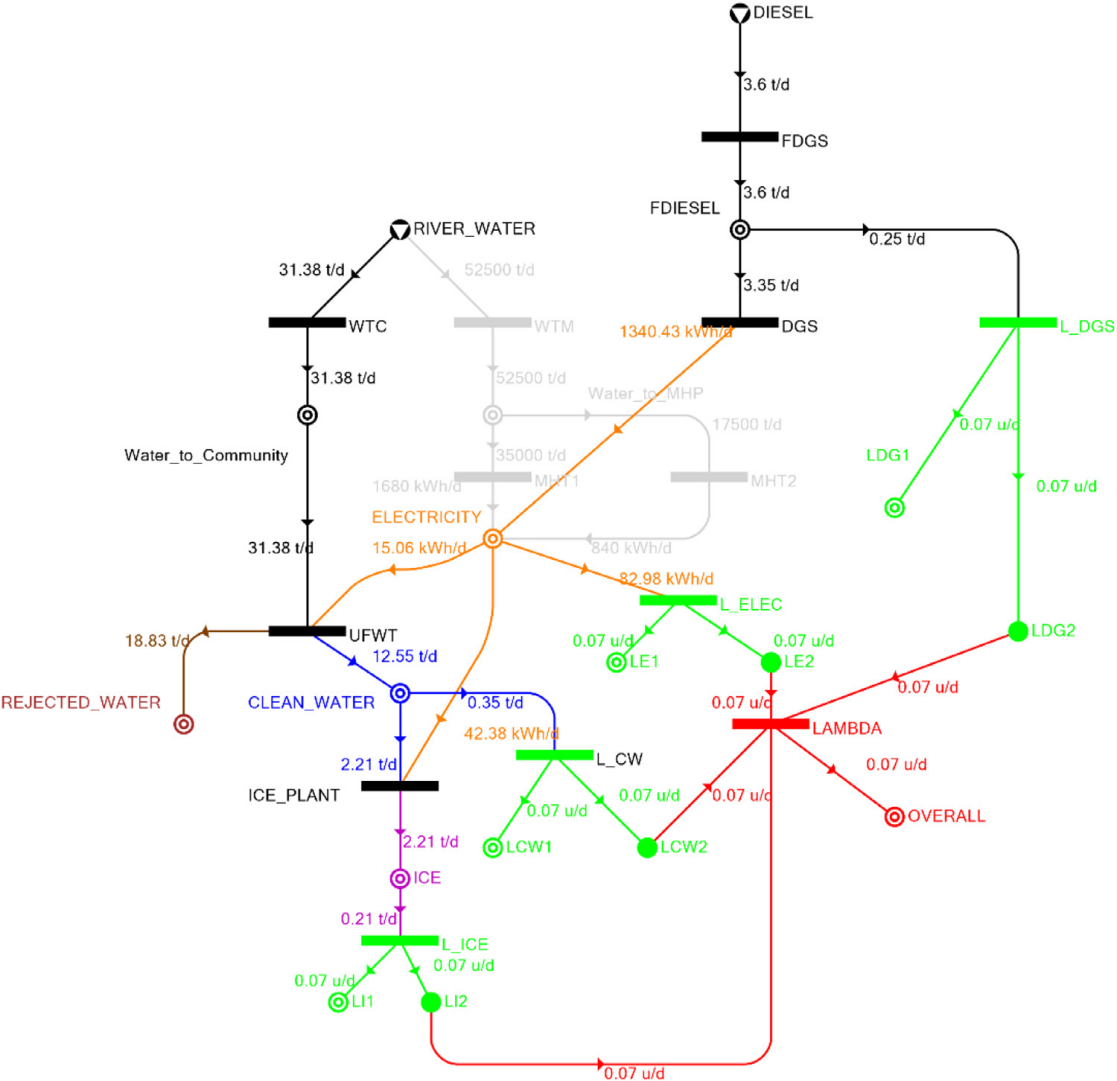
P-graph representation of micro-hydroelectric system at 90% drought level with Diesel Generator.

Table 2 shows the balanced process matrix for the off-grid community utility system during normal operating conditions and it is illustrated in P-graph form in Fig. 1. Table 3 summarizes the balanced process matrix when the drought level is at 10% and no diesel generator set is available, while Fig. 2 shows the equivalent P-graph form. Table 4 summarizes the balanced process matrix for a drought level of 20% (with no diesel generator set) with the corresponding P-graph representation in Fig. 3. Table 5 shows the balanced matrix for when the drought level is at 30% and Fig. 4 shows the equivalent P-graph representation. Table 6 shows the balanced matrix for when the system is at a drought level of 40% with its corresponding P-graph in Fig. 5. Table 7 shows the balanced process matrix for a drought level of 50% with no diesel generator back-up, Fig. 6 shows the P-graph representation corresponding to it. Table 8 shows the balanced process matrix for a 10% drought with available diesel generator back-up. The corresponding P-graph figure is shown in Fig. 7. Table 9 summarizes the balanced matrix for a 20% drought level with available diesel generator with the system illustrated in P-graph in Fig. 8. Table 10 shows the balanced matrix for a drought level of 30% when a diesel generator is available for back-up, the system is then illustrated in P-graph in Fig. 9. Table 11 contains the balanced process matrix at 40% drought level with an available Diesel Generator back-up. This is then illustrated in Fig. 10. Table 12 contains the balanced matrix for a drought level of 50% when a diesel generator is available for back-up, Fig. 11 illustrates the P-graph representation. Table 13 contains the balanced matrix for a 60% drought level and its corresponding P-graph representation is shown in Fig. 12. Table 14 contains the balanced process matrix at a drought level of 70% and is illustrated in P-graph representation in Fig. 13. Table 15 contains the balanced matrix for a drought level of 80% and is illustrated in P-graph form in Fig. 14. Table 16 contains the balanced matrix for a drought level of 90% and illustrated in Fig. 15.

**Table 2:** Balanced process matrix for 0% drought (over-all λ = 1.00)

Product Stream	Units	WTC	WTM	UFWT	Ice Plant	MHT1	MHT2	DGS	Product/Raw Material	λ
Clean water	t/d	0.00	0.00	20.00	−5.00	0.00	0.00	0.00	15	1.00
Ice	t/d	0.00	0.00	0.00	5.00	0.00	0.00	0.00	5	1.00
Electricity	kW	0.00	0.00	−1.00	−4.00	70.00	35.00	0.00	100	1.00
Water to community	t/d	50.00	0.00	−50.00	0.00	0.00	0.00	0.00	0	n/a
Water to MHP	t/d	0.00	52,500.00	0.00	0.00	−35,000.00	−17,500.00	0.00	0	n/a
Rejected water	t/d	0.00	0.00	30.00	0.00	0.00	0.00	0.00	30	n/a
River Water	t/d	−50.00	−52,500.00	0.00	0.00	0.00	0.00	0.00	−52,550	n/a
Diesel	t/d	0.00	0.00	0.00	0.00	0.00	0.00	0.00	0	1.00

**Table 3:** Balanced matrix for 10% drought level (over-all λ = 0.80)

Product Stream	Units	WTC	WTM	UFWT	Ice Plant	MHT1	MHT2	DGS	Product/Raw Material	λ
Clean water	t/d	0.00	0.00	18.41	−4.40	0.00	0.00	0.00	14.01	0.80
Ice	t/d	0.00	0.00	0.00	4.40	0.00	0.00	0.00	4.40	0.80
Electricity	kW	0.00	0.00	−0.92	−3.52	70.00	24.50	0.00	90.05	0.80
Water to community	t/d	46.02	0.00	−46.02	0.00	0.00	0.00	0.00	0.00	n/a
Water to MHP	t/d	0.00	47,249.00	0.00	0.00	−35000.00	−12,249.00	0.00	0.00	n/a
Rejected water	t/d	0.00	0.00	27.61	0.00	0.00	0.00	0.00	27.61	n/a
River Water	t/d	−46.02	−47,249.00	0.00	0.00	0.00	0.00	0.00	−47,295.02	n/a
Diesel	t/d	0.00	0.00	0.00	0.00	0.00	0.00	0.00	0.00	1.00

**Table 4:** Balanced matrix for 20% drought level (over-all λ = 0.60)

Product Stream	Units	WTC	WTM	UFWT	Ice Plant	MHT1	MHT2	DGS	Product/Raw Material	λ
Clean water	t/d	0.00	0.00	16.82	−3.81	0.00	0.00	0.00	13.01	0.60
Ice	t/d	0.00	0.00	0.00	3.81	0.00	0.00	0.00	3.81	0.60
Electricity	kW	0.00	0.00	−0.84	−3.05	68.25	15.75	0.00	80.11	0.60
Water to community	t/d	42.05	0.00	−42.05	0.00	0.00	0.00	0.00	0.00	n/a
Water to MHP	t/d	0.00	41,997.95	0.00	0.00	−34,122.95	−7,875.00	0.00	0.00	n/a
Rejected water	t/d	0.00	0.00	25.23	0.00	0.00	0.00	0.00	25.23	n/a
River Water	t/d	−42.05	−41,997.95	0.00	0.00	0.00	0.00	0.00	−42,040.00	n/a
Diesel	t/d	0.00	0.00	0.00	0.00	0.00	0.00	0.00	0.00	1.00

**Table 5:** Balanced matrix for 30% drought level (over-all λ = 0.40)

Product Stream	Units	WTC	WTM	UFWT	Ice Plant	MHT1	MHT2	DGS	Product/Raw Material	λ
Clean water	t/d	0.00	0.00	15.23	−3.21	0.00	0.00	0.00	12.02	0.40
Ice	t/d	0.00	0.00	0.00	3.21	0.00	0.00	0.00	3.21	0.40
Electricity	kW	0.00	0.00	−0.76	−2.57	57.74	15.75	0.00	70.16	0.40
Water to community	t/d	38.07	0.00	−38.07	0.00	0.00	0.00	0.00	0.00	n/a
Water to MHP	t/d	0.00	36,746.93	0.00	0.00	−28,871.93	−7,875.00	0.00	0.00	n/a
Rejected water	t/d	0.00	0.00	22.84	0.00	0.00	0.00	0.00	22.84	n/a
River Water	t/d	−38.07	−36,746.96	0.00	0.00	0.00	0.00	0.00	−36,785.02	n/a
Diesel	t/d	0.00	0.00	0.00	0.00	0.00	0.00	0.00	0.00	1.00

**Table 6:** Balanced matrix for 40% drought level (over-all λ = 0.20)

Product Stream	Units	WTC	WTM	UFWT	Ice Plant	MHT1	MHT2	DGS	Product/Raw Material	λ
Clean water	t/d	0.00	0.00	13.64	−2.61	0.00	0.00	0.00	11.03	0.20
Ice	t/d	0.00	0.00	0.00	2.61	0.00	0.00	0.00	2.61	0.20
Electricity	kW	0.00	0.00	−0.68	−2.09	62.99	0.00	0.00	60.22	0.20
Water to community	t/d	34.09	0.00	−34.09	0.00	0.00	0.00	0.00	0.00	n/a
Water to MHP	t/d	0.00	31,495.91	0.00	0.00	−31,495.91	0.00	0.00	0.00	n/a
Rejected water	t/d	0.00	0.00	20.45	0.00	0.00	0.00	0.00	20.45	n/a
River Water	t/d	−34.09	−31,495.91	0.00	0.00	0.00	0.00	0.00	−31,530.00	n/a
Diesel	t/d	0.00	0.00	0.00	0.00	0.00	0.00	0.00	0.00	1.00

**Table 7:** Balanced matrix for 50% drought level (over-all λ = 0.005)

Product Stream	Units	WTC	WTM	UFWT	Ice Plant	MHT1	MHT2	DGS	Product/Raw Material	λ
Clean water	t/d	0.00	0.00	12.04	−2.02	0.00	0.00	0.00	10.02	0.005
Ice	t/d	0.00	0.00	0.00	2.02	0.00	0.00	0.00	2.02	0.005
Electricity	kW	0.00	0.00	−0.60	−1.61	52.49	0.00	0.00	50.27	0.005
Water to community	t/d	30.11	0.00	−30.11	0.00	0.00	0.00	0.00	0.00	n/a
Water to MHP	t/d	0.00	26,244.91	0.00	0.00	−26,244.91	0.00	0.00	0.00	n/a
Rejected water	t/d	0.00	0.00	18.07	0.00	0.00	0.00	0.00	18.07	n/a
River Water	t/d	−30.11	−26,244.91	0.00	0.00	0.00	0.00	0.00	−26,275.02	n/a
Diesel	t/d	0.00	0.00	0.00	0.00	0.00	0.00	0.00	0.00	1.00

**Table 8:** Balanced matrix for 10% drought level with Diesel Generator (over-all λ = 0.91)

Product Stream	Units	WTC	WTM	UFWT	Ice Plant	MHT1	MHT2	DGS	Product/Raw Material	λ
Clean water	t/d	0.00	0.00	19.25	−4.72	0.00	0.00	0.00	14.53	0.91
Ice	t/d	0.00	0.00	0.00	4.72	0.00	0.00	0.00	4.72	0.91
Electricity	kW	0.00	0.00	−0.96	−3.78	70.00	24.49	5.59	95.34	0.91
Water to community	t/d	48.14	0.00	−48.14	0.00	0.00	0.00	0.00	0.00	n/a
Water to MHP	t/d	0.00	47,246.85	0.00	0.00	−35,000.00	−12,246.85	0.00	0.00	n/a
Rejected water	t/d	0.00	0.00	28.88	0.00	0.00	0.00	0.00	28.88	n/a
River Water	t/d	−48.15	−47,246.85	0.00	0.00	0.00	0.00	0.00	−47,295.00	n/a
Diesel	t/d	0.00	0.00	0.00	0.00	0.00	0.00	−0.34	−0.34	1.00

**Table 9:** Balanced matrix for 20% drought level with Diesel Generator (over-all λ = 0.81)

Product Stream	Units	WTC	WTM	UFWT	Ice Plant	MHT1	MHT2	DGS	Product/Raw Material	λ
Clean water	t/d	0.00	0.00	18.51	−4.44	0.00	0.00	0.00	14.07	0.81
Ice	t/d	0.00	0.00	0.00	4.44	0.00	0.00	0.00	4.44	0.81
Electricity	kW	0.00	0.00	−0.93	−3.55	68.24	15.75	11.18	90.69	0.81
Water to community	t/d	46.27	0.00	−46.27	0.00	0.00	0.00	0.00	0.00	n/a
Water to MHP	t/d	0.00	41,993.70	0.00	0.00	−34,118.74	−7,875.00	0.00	−0.03	n/a
Rejected water	t/d	0.00	0.00	27.76	0.00	0.00	0.00	0.00	27.76	n/a
River Water	t/d	−46.27	−41,993.70	0.00	0.00	0.00	0.00	0.00	−42,039.97	n/a
Diesel	t/d	0.00	0.00	0.00	0.00	0.00	0.00	−0.67	−0.67	0.81

**Table 10:** Balanced matrix for 30% drought level with Diesel Generator (over-all λ = 0.72)

Product Stream	Units	WTC	WTM	UFWT	Ice Plant	MHT1	MHT2	DGS	Product/Raw Material	λ
Clean water	t/d	0.00	0.00	17.76	−4.16	0.00	0.00	0.00	13.60	0.72
Ice	t/d	0.00	0.00	0.00	4.16	0.00	0.00	0.00	4.16	0.72
Electricity	kW	0.00	0.00	−0.89	−3.33	57.73	15.75	16.77	86.03	0.72
Water to community	t/d	44.41	0.00	−44.41	0.00	0.00	0.00	0.00	0.00	n/a
Water to MHP	t/d	0.00	36,740.60	0.00	0.00	−28,865.60	−7,875.00	0.00	0.00	n/a
Rejected water	t/d	0.00	0.00	26.65	0.00	0.00	0.00	0.00	26.65	n/a
River Water	t/d	−44.41	−36,740.60	0.00	0.00	0.00	0.00	0.00	−36,785.01	n/a
Diesel	t/d	0.00	0.00	0.00	0.00	0.00	0.00	−1.01	−1.01	0.72

**Table 11:** Balanced matrix for 40% drought level with Diesel Generator (over-all λ = 0.63)

Product Stream	Units	WTC	WTM	UFWT	Ice Plant	MHT1	MHT2	DGS	Product/Raw Material	λ
Clean water	t/d	0.00	0.00	17.02	−3.88	0.00	0.00	0.00	13.14	0.63
Ice	t/d	0.00	0.00	0.00	3.88	0.00	0.00	0.00	3.88	0.63
Electricity	kW	0.00	0.00	−0.85	−3.11	62.97	0.00	22.35	81.37	0.63
Water to community	t/d	42.55	0.00	−42.55	0.00	0.00	0.00	0.00	0.00	n/a
Water to MHP	t/d	0.00	31,487.45	0.00	0.00	−31,487.44	0.00	0.00	0.02	n/a
Rejected water	t/d	0.00	0.00	25.53	0.00	0.00	0.00	0.00	25.53	n/a
River Water	t/d	−42.55	−31,487.45	0.00	0.00	0.00	0.00	0.00	−31,530.00	n/a
Diesel	t/d	0.00	0.00	0.00	0.00	0.00	0.00	−1.34	−1.34	0.63

**Table 12:** Balanced matrix for 50% drought level with Diesel Generator (over-all λ = 0.53)

Product Stream	Units	WTC	WTM	UFWT	Ice Plant	MHT1	MHT2	DGS	Product/Raw Material	λ
Clean water	t/d	0.00	0.00	16.27	−3.60	0.00	0.00	0.00	12.67	0.53
Ice	t/d	0.00	0.00	0.00	3.60	0.00	0.00	0.00	3.60	0.53
Electricity	kW	0.00	0.00	−0.81	−2.88	52.47	0.00	27.94	76.71	0.53
Water to community	t/d	40.69	0.00	−40.69	0.00	0.00	0.00	0.00	0.00	n/a
Water to MHP	t/d	0.00	26,234.30	0.00	0.00	−26,234.30	0.00	0.00	0.00	n/a
Rejected water	t/d	0.00	0.00	24.41	0.00	0.00	0.00	0.00	24.41	n/a
River Water	t/d	−40.69	−26,234.30	0.00	0.00	0.00	0.00	0.00	−26,274.99	n/a
Diesel	t/d	0.00	0.00	0.00	0.00	0.00	0.00	−1.68	−1.68	0.53

**Table 13:** Balanced matrix for 60% drought level (over-all λ = 0.44)

Product Stream	Units	WTC	WTM	UFWT	Ice Plant	MHT1	MHT2	DGS	Product/Raw Material	λ
Clean water	t/d	0.00	0.00	15.53	−3.32	0.00	0.00	0.00	12.21	0.44
Ice	t/d	0.00	0.00	0.00	3.32	0.00	0.00	0.00	3.32	0.44
Electricity	kW	0.00	0.00	−0.78	−2.66	41.96	0.00	33.53	72.06	0.44
Water to community	t/d	38.82	0.00	−38.82	0.00	0.00	0.00	0.00	0.00	n/a
Water to MHP	t/d	0.00	20,981.15	0.00	0.00	−20,981.15	0.00	0.00	0.00	n/a
Rejected water	t/d	0.00	0.00	23.29	0.00	0.00	0.00	0.00	23.29	n/a
River Water	t/d	−38.82	−20,981.15	0.00	0.00	0.00	0.00	0.00	−21,019.98	n/a
Diesel	t/d	0.00	0.00	0.00	0.00	0.00	0.00	−2.01	−2.01	0.44

**Table 14:** Balanced matrix for 70% drought level (over-all λ = 0.35)

Product Stream	Units	WTC	WTM	UFWT	Ice Plant	MHT1	MHT2	DGS	Product/Raw Material	λ
Clean water	t/d	0.00	0.00	14.78	−3.04	0.00	0.00	0.00	11.74	0.35
Ice	t/d	0.00	0.00	0.00	3.04	0.00	0.00	0.00	3.04	0.35
Electricity	kW	0.00	0.00	−0.74	−2.44	0.00	31.46	39.12	67.40	0.35
Water to community	t/d	36.96	0.00	−36.96	0.00	0.00	0.00	0.00	0.00	n/a
Water to MHP	t/d	0.00	15,728.06	0.00	0.00	0.00	−15,728.06	0.00	0.00	n/a
Rejected water	t/d	0.00	0.00	22.18	0.00	0.00	0.00	0.00	22.18	n/a
River Water	t/d	−36.96	−15,728.06	0.00	0.00	0.00	0.00	0.00	−15,765.02	n/a
Diesel	t/d	0.00	0.00	0.00	0.00	0.00	0.00	−2.35	−2.35	0.35

**Table 15:** Balanced matrix for 80% drought level (over-all λ = 0.25)

Product Stream	Units	WTC	WTM	UFWT	Ice Plant	MHT1	MHT2	DGS	Product/Raw Material	λ
Clean water	t/d	0.00	0.00	14.04	−2.76	0.00	0.00	0.00	11.27	0.25
Ice	t/d	0.00	0.00	0.00	2.76	0.00	0.00	0.00	2.76	0.25
Electricity	kW	0.00	0.00	−0.70	−2.21	0.00	20.95	44.71	62.74	0.25
Water to community	t/d	35.10	0.00	−35.10	0.00	0.00	0.00	0.00	0.00	n/a
Water to MHP	t/d	0.00	10,474.91	0.00	0.00	0.00	−10,474.91	0.00	0.00	n/a
Rejected water	t/d	0.00	0.00	21.06	0.00	0.00	0.00	0.00	21.06	n/a
River Water	t/d	−35.10	−10,474.91	0.00	0.00	0.00	0.00	0.00	−10,510.00	n/a
Diesel	t/d	0.00	0.00	0.00	0.00	0.00	0.00	−2.35	−2.35	0.25

**Table 16:** Balanced matrix for 90% drought level (over-all λ = 0.07)

Product Stream	Units	WTC	WTM	UFWT	Ice Plant	MHT1	MHT2	DGS	Product/Raw Material	λ
Clean water	t/d	0.00	0.00	12.55	−2.21	0.00	0.00	0.00	10.35	0.07
Ice	t/d	0.00	0.00	0.00	2.21	0.00	0.00	0.00	2.21	0.07
Electricity	kW	0.00	0.00	−0.63	−1.77	0.00	0.00	55.85	53.46	0.07
Water to community	t/d	31.38	0.00	−31.38	0.00	0.00	0.00	0.00	0.00	n/a
Water to MHP	t/d	0.00	0.00	0.00	0.00	0.00	0.00	0.00	0.00	n/a
Rejected water	t/d	0.00	0.00	18.83	0.00	0.00	0.00	0.00	18.83	n/a
River Water	t/d	−31.38	0.00	0.00	0.00	0.00	0.00	0.00	−31.38	n/a
Diesel	t/d	0.00	0.00	0.00	0.00	0.00	0.00	−3.35	−3.35	0.07

## 2 Experimental Design, Materials, and Methods

The system was subjected to 16 scenarios of drought conditions where the level of available water resources was decreased in increments of 10% from 100% (for the baseline scenario) to 0% in cases of extreme drought and in consideration of (1) no back-up diesel generator system and (2) with back-up diesel generator system. The summary of the conditions for each scenario can be found in Table 1. The process matrix for the system considered can be found in Aviso et al. [1] together with the product demand.

## Declaration of Competing Interest

The authors declare that they have no known competing financial interests or personal relationships which have, or could be perceived to have, influenced the work reported in this article.
